# Racial Discrimination and Mental Health Among Latine Adolescents: Parenting and Conflict as Potential Moderators

**DOI:** 10.1007/s40615-026-02956-6

**Published:** 2026-04-07

**Authors:** Haleigh Kampman, Matthew Aalsma, Patrick Monahan, Natalie Guerrero

**Affiliations:** 1 Department of Health Policy and Management, Indiana University Richard M. Fairbanks School of Public Health, Indianapolis, IN, USA; 2 Regenstrief Center for Health Equity Research, Eskenazi Health, Indianapolis, IN, USA; 3 Department of Pediatrics, Indiana University School of Medicine, Indianapolis, IN, USA; 4 Department of Biostatistics and Health Data Science, Indiana University School of Medicine, Indianapolis, IN, USA

## Abstract

**Objectives:**

Our study aimed to understand whether parenting practices, including positive parenting and caregiver involvement, and family conflict, moderated the relationship between experiences of racial-ethnic discrimination and mental health outcomes among Latine adolescents.

**Methods:**

We used a cross-sectional study design using surveys administered to Latine adolescents ages 13–17 years and their primary caregivers in Indianapolis, Indiana. Data collected included demographics, experiences of racial-ethnic discrimination measured by the Everyday Discrimination Scale (EDS), conflict (Parent-Child Conflict) and parenting (Parenting Practices), symptoms of anxiety (Generalized Anxiety Disorder - GAD-7), depression (Mood and Feelings Questionnaire - MFQ), and internalizing and externalizing symptoms (Strengths and Difficulties Questionnaire – SDQ). Data were analyzed using multivariable linear regressions. Interaction terms were added to these models to test whether parenting practices (positive parenting and caregiver involvement), and caregiver-adolescent conflict, moderated the relationship between adolescents’ experiences of racial-ethnic discrimination and measures of mental health.

**Results:**

Of 127 caregiver-adolescent dyads that responded to the survey, 92 were included in the analyses. Our sample included mostly female adolescents (60.9%) whose mean age was 15 years old. Regression analyses confirmed the negative effects of racial-ethnic discrimination on adolescent’s mental health outcomes. Positive parenting was consistently identified as having a positive effect on mental health. No moderating effects were identified.

**Conclusions:**

Our results have confirmed that racial-ethnic discrimination has poor implications for Latine adolescents’ mental health, while positive parenting has mental health-promotive effects. However, targeted interventions that build caregivers’ skills, beyond positive parenting, in creating a nurturing and supportive environment for their child may be needed.

## Introduction

The prevalence of anxiety [1, 2], depression [2–4], and more severe outcomes, like suicide [5, 6], have rapidly increased among the youth and adolescent populations. In a recent study, adolescents aged 14–17 reported the highest rates of depression and anxiety compared to other age groups [2]. Historically marginalized populations (i.e., Hispanic/Latine) are diagnosed with mental health disorders at disproportionately higher rates [7–9]. This may be, in part, a result of racial-ethnic discrimination, a known psychosocial stressor with negative impacts on adolescent mental health [10–12].

Recently, Lorenzo-Blanco and colleagues [10] described how Hispanic/Latine adolescents cope with racism and cultural stressors. Adolescents distanced themselves from discrimination, attempted to restore their emotional balance by seeking companionship, or confronted/boycotted discriminatory situations, sometimes interpreted as disruptive or aggressive behaviors [10]. If they were unable to engage in these coping mechanisms, they often sought emotional support from others, attempted to suppress emotions (i.e., internalizing behaviors), or, at times, isolated themselves from their support network [10].

Given the role racial-ethnic discrimination plays in mental health, it is increasingly important that a greater understanding of the role of parenting practices (e.g., encouraging children, being a positive role model) including positive parenting/involvement (e.g., attending outings together), and caregiver-teen conflict. Positive parenting and involvement may be important targets for intervention, as they are documented as one of the strongest predictors of healthy child and adolescent development, and function as protective factors against negative or stressful experiences an adolescent may encounter [11, 12].

Conversely, caregiver-adolescent conflict may be detrimental to adolescent development and is associated with worse adolescent mental health [13] and emotional distress [14]. One contributor that may result in poor family dynamics is the differing rate at which acculturation, or the process at which populations adapt to new behaviors and beliefs, occurs between adolescents and their caregivers. Typically, adolescents socialize at higher frequencies in a new country (e.g., going to school or participating in extracurricular activities) and tend to acculturate faster than their caregivers, resulting in conflict. Family dynamics (i.e., conflict) may be another point of intervention to promote mental health among Latine adolescents who have experienced racial-ethnic discrimination.

While prior studies [15, 16] have described the direct relationship between parenting practices and conflict in Latine adolescent mental health, few have examined [20–21] moderating effects [17–18] of these factors, which can offer a more nuanced view of how these may impact the relationship between racial-ethnic discrimination and mental health outcomes. Our study examined whether measures of parenting practices and caregiver-adolescent conflict, moderated the association between racial-ethnic discrimination experiences and mental health among Latine adolescents, namely anxiety, depression, and internalizing and externalizing symptoms. Results of this study can aid in the development or refinement of existing caregiver-adolescent interventions promoting Latine adolescent mental health. We hypothesized that (1) caregiver-adolescent conflict would exacerbate the relationship between racial-ethnic discrimination and mental health, and (2) positive parenting and separately, (3) caregiver involvement would buffer this relationship.

## Methods

### Sample

We conducted a cross-sectional study using survey data from *Project Salud*, a mixed methods dyadic study that examined the role of family dynamics, experiences of racial-ethnic discrimination, and mental health among Latine adolescents and their caregivers. Participants were recruited via paper and electronic flyers in English and Spanish, and were distributed at schools, community organizations, and churches in Indianapolis, Indiana. Research team members followed up with interested individuals via email to confirm interest and eligibility. Adolescents and caregivers received information about the study and provided caregiver consent and adolescent assent in their preferred language (English or Spanish). To be eligible: (1) adolescents must have been between 13 and 17 years old, (2) adolescents and caregivers must have identified as Hispanic/Latine, (3) both must have been fluent in English or Spanish, and (4) both must have provided assent/consent to participate.

Surveys, which included the same measures for both the caregiver and adolescent, were administered via personalized links sent via email to consented/assented participants between April and August 2024. Data were collected in the participant’s preferred language using REDCap (Research Electronic Data Capture) [19–20] a secure, web-based platform, designed for administering and managing electronic surveys. The survey took approximately 60 min to complete, and participants received a $50 gift card as compensation. This study was approved by the Indiana University Institutional Review Board (Protocol #16925).

### Measures

#### Demographics

The adolescent’s self-reported demographic characteristics, including the age, sex assigned at birth, Latino/a race (White, Black, Asian, Indigenous, Other), preferred language, and country of birth were included. Socioeconomic status was captured using the caregiver’s response to the question, “Last month, what was the total amount of money you and your family lived on, including public assistance (after taxes)?” Response options included “less than $200”, “$200-$499”, “$500-$999”, “1000-$1999”, and “2000 or more”. In our analyses, response options of “less than $200” and “$200-$499” were combined due to small cell size.

#### Conflict

Parent-adolescent Conflict, a 15-item scale, was used to assess the level of conflict the adolescent perceived with the caregiver who participated in the survey [21]. The measure has been adapted for use with Mexican-American and Latino families [22]. Items ask how often adolescents disagreed with their caregiver about various topics, including: money, curfew, choice of friends, chores, privacy, etc. Six response options were available for each question, including: “not at all”, “a couple of times”, “a few times each month”, “several times each week”, “about once a day”, or “several times a day”. Responses were then assigned a value from 1 to 6, with higher scores indicative of more conflict. All items were averaged to create a total score.

#### Parenting Practices

Parental practices of positive parenting and involvement were assessed using two subscales from the 17-item questionnaire [23], positive parenting subscale and the extent of parental involvement in the adolescent’s life subscale. This questionnaire has been adapted for Spanish-speaking populations with various dialects [24]. Response options for each item are “never”, “hardly ever”, “sometimes”, “usually”, and “always” and were coded 1–5, respectively. All scores reported by the adolescent were averaged for each subscale.

#### Racial-Ethnic Discrimination

Racial-ethnic discrimination was assessed using the Everyday Discrimination Scale [25] and has been validated in Spanish-speaking populations [26]. This scale includes nine questions and asks how often the adolescent has experienced discriminatory situations. Because Project Salud was specifically interested in the impacts of racial-ethnic discrimination experiences, adolescents were asked how often these nine discrimination experiences occurred “because of your race or ethnicity.” Response options include “almost every day”, “at least once a week”, “a few times a month”, “a few times a year”, “less than once a year”, and “never”. Responses were coded from “0” to “5” with “0” representing “never” and “5” representing “almost every day” and summed to create a total score that ranged from 0-45.

#### Anxiety

Anxiety was measured using the Generalized Anxiety Disorder Assessment (GAD-7) [27], a seven-item questionnaire used to assess adolescents’ severity of their anxiety over the past two weeks. Responses across the seven questions were summed to create a total score, ranging from 0 to 21, with higher scores indicating higher severity of anxiety symptoms. The GAD-7 has been studied in Spanish-speaking populations, demonstrating validity [28].

#### Internalizing and Externalizing Symptoms

The Strengths and Difficulties Questionnaire (SDQ) is a 25-item instrument comprised of five subscales, each with 5 items, to assess emotional and behavioral difficulties in adolescents [29]. The subscales include questions assessing emotional problems, conduct problems, hyperactivity, peer problems, and prosocial behavior. Available responses include “not true”, “somewhat true”, and “certainly true” for each item. The SDQ has been validated among adolescents aged 11–16 years old [29], and Spanish-speaking populations [30]. Responses were summed for each subscale except for prosocial behavior to generate a total difficulties score ranging from 0 to 40. Higher scores indicated a greater number of difficulties.

#### Depressive Symptoms

The Mood and Feelings Questionnaire (MFQ) is a 33-item instrument to assess depressive symptoms in adolescents and adults [31]. It has demonstrated reliability and validity in research and practice among youth 6–17 years old [31]. Response options include “not true,” “sometimes,” and “true” for each question. Scores were summed across all questions, resulting in a range from 0 to 26. Higher scores indicated greater depressive symptomology. The MFQ includes suicidality items, and if the adolescent endorsed any of these items, the research team received an automated notification via email. The team then evaluated the adolescent’s responses for suicidality and conducted a follow-up phone call with the caregiver within 24 h to discuss appropriate care options and provide relevant resources. If the caregiver could not be reached by phone, a follow-up email was sent with detailed information and available resources to support the adolescent’s care.

The MFQ, SDQ, GAD-7, the Everyday Discrimination Scale, parenting practices, and parent-adolescent conflict scales have demonstrated good reliability and validity and previous studies. Additional details can be found in Appendix A.

### Statistical Analyses

Descriptive statistics, including counts and frequencies, were used to characterize adolescents’ responses. We employed linear regression analyses using adolescents’ completed survey responses to examine how measures of parenting practices and conflict moderated the effect of racial-ethnic discrimination and mental health. For each mental health outcome (GAD-7, SDQ, and MFQ), four regression analyses were run, each adjusting for demographic characteristics including the adolescent’s age, sex assigned at birth, and the caregiver’s income as a proxy for socioeconomic status. Our first model included racial-ethnic discrimination, family conflict, positive parenting, and caregiver involvement. Models 2–4 included one of the three potential moderators of interest (positive parenting, involvement, or conflict) along with its corresponding interaction term between the moderator of interest and racial-ethnic discrimination. For example, Model 2 included demographics, racial-ethnic discrimination, family conflict, and an interaction term for family conflict and racial-ethnic discrimination. Similarly, Model 3 included demographics, racial-ethnic discrimination, positive parenting, and the interaction between positive parenting and racial-ethnic discrimination. Because sex was a categorical variable that included response options of male or female, male was the reference group in regression analyses. All tests were two-sided, and results were considered statistically significant at a p-value < 0.05. Given the exploratory nature of this study, corrections for multiple comparisons were not applied. All analyses were conducted in RStudio Version 4.4.3. We also conducted post hoc power analyses to evaluate the minimum sample size required to detect significant effect sizes.

## Results

Of the 127 dyads (adolescents and their caregivers) who responded to the survey, 92 adolescents were included in our analyses. Excluded participants included adolescents who reported being outside the ages of 13–17 years old when they submitted the survey (*n* = 2) and whose responses were incomplete (*n* = 35); details are shown in Appendix B. Comparing the characteristics between participants included and excluded, those excluded tended to report Spanish as their preferred language (*n* = 14, 40%) at a higher percentage, as compared to those included (*n* = 26, 28%). Additionally, those excluded had slightly higher reports of being born in Mexico (*n* = 5, 14%) or Honduras (*n* = 3, 9%). Table 1 presents characteristics of the adolescents included. Most (*n* = 56, 60.9%) adolescents were female, with a mean age of 15 years old, and identified their race as White Latino/a (*n* = 58, 63.0%). Approximately three-quarters of the adolescent sample indicated their preferred language as English (*n* = 66, 71.7%) and were born in the United States (*n* = 70, 76.1%).

In multivariable regression analyses shown in Table 2 (Models 1, 5, and 9), we identified that higher scores of racial-ethnic discrimination were significantly associated with higher scores on all three mental health measures. We also identified a statistically significant inverse relationship between scores on the positive parenting subscale and each mental health outcome; lower scores for positive parenting were associated with higher scores on the GAD-7, SDQ, and MFQ, or worse mental health. Specifically, a one-point increase in positive parenting was associated with a 2.5 point reduction on the GAD-7, 1.6 points on the SDQ, and 4.4 points on the MFQ, suggesting meaningful improvements in adolescent mental health. Lastly, scores for family conflict were significantly positively associated with SDQ and MFQ scores, but not GAD-7 scores.

Models 2–4, 6–8, and 10–12, which included interaction terms to test moderating effects, were not statistically significantly associated with any of the outcomes. Furthermore, models with an interaction term between family conflict and racial-ethnic discrimination (Models 2, 6, and 10) had attenuated the effect size of family conflict as compared to models with no moderating effects (Models 1, 5, and 9). For the SDQ and MFQ outcomes specifically, the significance present for family conflict (models 5 and 9) was no longer present after the addition of the interaction term (models 6 and 10). Conversely, Models 3, 7, and 11, which included an interaction between positive parenting and racial discrimination, maintained their significance on the positive parenting variable (as compared to models without the interaction (1, 5, and 9). Lastly, despite caregiver involvement having no moderating effect across all models, model 8 showed a statistically significant inverse relationship between caregiver involvement (β = −2.6, *p*<.001) and SDQ scores, suggesting that lower levels of caregiver involvement may have an effect on externalizing and internalizing symptoms.

## Discussion

Latine adolescents, an often-understudied population [32], commonly experience racial-ethnic discrimination, which is known to have detrimental effects on mental health [33–38]. This study investigated whether parenting practices, including caregiver involvement and positive parenting, and caregiver-adolescent conflict, as perceived by adolescents, moderated the relationship between experiences of racial-ethnic discrimination and mental health outcomes. By examining both direct and moderating effects, our analyses offer a more nuanced understanding of the roles that parenting practices and conflict may play in the context of racial-ethnic discrimination.

Our findings that higher levels of discrimination were significantly and positively associated with anxiety, depression, and internalizing and externalizing scores confirmed the harmful and widespread impact of racial-ethnic discrimination on Latine adolescents’ mental health. Discrimination was associated with heightened anxiety symptomology, such as excessive worry and feelings of agitation, consistent with existing literature [39]. Similarly, our findings showed a positive association between discrimination and MFQ scores, representing more frequent feelings of internalizing symptoms of depression, loss of interest, poor self-esteem, and suicidal ideation [31]. Internalizing symptoms can be related to externalizing symptoms as well. For example, uncontrolled anxiety oftentimes triggers the “fight or flight” response, resulting in aggressive behaviors, arguments, and/or lashing out [38, 40]. In our study, SDQ scores were also positively associated with higher reports of discrimination. Our findings are consistent with the literature and suggest that the impacts of discrimination may extend beyond internalizing symptoms, impacting externalizing symptoms as well [10, 35]. To help decrease exposure to racial-ethnic discrimination at a young age, a multi-faceted approach would be required in communities. One strategy to mitigate the effects of discrimination is prioritizing the recruitment and hiring of individuals, in organizations that adolescents frequent, whose backgrounds reflect the populations being served. Furthermore, because adolescents spend a substantial portion of their time in school settings, incorporating more inclusive curricula that expose students to diverse histories and cultures may help promote greater understanding and reduce bias.

Our analyses also highlighted the mental health-promoting role of positive parenting. Adolescents who perceived their caregivers as supportive, responsive, and communicative reported lower scores on anxiety, depression, and behavioral difficulties. These findings align with prior research suggesting that a nurturing home environment, characterized by emotional support and open communication, can help promote mental health [9, 11, 41–43]. As such, strengthening positive parenting practices may be a key focus for interventions aimed at improving mental health outcomes among Latine adolescents.

While positive parenting was found to predict all of the mental health outcomes we measured, conflict and caregiver involvement were not consistent predictors of mental health. For example, our results showed that caregiver-adolescent conflict predicted adolescents’ higher internalizing and externalizing symptoms, and depressive symptoms, yet did not play a role in symptoms of anxiety. Additionally, caregiver involvement was found to have few associations with mental health outcomes. This may suggest that, at the adolescent stage of development, children may be seeking companionship outside the formal parent-caregiver structure and may be more reliant on social support provided by peers. This may provide an opportunity to assist adolescents in developing these relationships in preventive intervention development.

Contrary to our hypotheses, parenting practices did not significantly moderate the relationship between racial-ethnic discrimination and mental health. In other words, these variables did not alter the association between adolescents’ experiences of discrimination and their self-reported mental health. Because adolescence is a period of increasing autonomy [44], parenting practices and family conflict may be less influential in how youth respond to racial-ethnic discrimination. Another explanation is that differences in appearance or cultural practices between Latine adolescents and their caregivers may shape the type or extent of support caregivers can provide. Strengthening formal and informal support structures within and beyond the family may help mitigate the long-term consequences of discrimination. Parental practices and low family conflict alone may be insufficient to buffer its negative effects. Social support networks that include non-family members could represent an additional protective mechanism and an important direction for future research.

Research has indicated that emotional support from parents and caregivers can play a strong role in mitigating effects of discrimination [10, 44], and another possibility of our null moderation results may be that our measurements for parenting practices and conflict were not sufficiently capturing how Latine families respond to racial-ethnic discrimination. Future research could evaluate how caregiver practices and relationships moderate the relationship between discrimination and mental health using measures that better capture this construct, specifically among Latine families. Namely, practices related to racial-ethnic socialization (RES), or the process by which adolescents learn and understand values and perspectives about race and ethnicity [33, 45–46] may be a key element of protection for Latine families. Although much of the literature on RES has focused on Black families [45], a growing literature on RES among Latine families is being established and has demonstrated promising findings in the healthy psychological development of adolescents [47–48]. Further research should be conducted to better understand the protective role of specific parenting practices and relationships and explore how Latine values and belief systems shape mental health.

## Limitations

While our study adds valuable evidence to current literature, we also note limitations of our study. First, our sample size was relatively small, and our findings were based on a convenience sample of Latine adolescents and caregivers living in Indianapolis, Indiana. This limits generalizability outside this setting and population. Additionally, though our sample size was sufficient to detect large effect sizes, our study did not have adequate power to detect smaller associations which may be clinically meaningful. Because we did not correct for multiple comparisons in our study, it is possible that statistically significant findings are due to chance, and as a result, findings should be interpreted with caution. Future research should replicate these analyses using a larger sample size.

## Conclusion

Our findings contribute evidence to the wide-ranging implications that racial-ethnic discrimination has on Latine adolescents’ wellbeing and may demonstrate a level of urgency to identify evidence-based interventions that mitigate these harmful experiences. Positive parenting may positively impact Latine mental health outcomes but may not be sufficient to buffer the negative impacts of racial-ethnic discrimination on Latine adolescent mental health. Developing targeted interventions that help Latine caregivers foster a positive and supportive environment that promotes positive racial-ethnic identity, and promotes supportive peer and other relationships, may together minimize the negative impacts of racial-ethnic discrimination on Latine adolescent mental health.

## Supplementary Material

Appendix A

Appendix B

The online version contains supplementary material available at https://doi.org/10.1007/s40615-026-02956-6.

## Figures and Tables

**Table 1:** Study population characteristics. Alt Text. Table detailing the adolescent’s age, sex, race, preferred language, and country born

	Adolescents *n* = 92
Age (mean, SD)	15.0 (1.5)
Sex (Female (n, %))	56 (60.9%)
Latino Race^1^	
White (Latino/a)	58 (63.0%)
Black-Latino/a (Afro-Latino/a)	7 (7.6%)
Asian-Latino/a	0 (0.0%)
Indigenous Latino/a	19 (20.6%)
Other	8 (8.7%)
Preferred Language	
English	66 (71.7%)
Spanish	26 (28.3%)
Indigenous language	0 (0.0%)
Country Born	
USA	70 (76.1%)
Mexico	8 (8.7%)
Honduras	5 (5.4%)
Venezuela	3 (3.3%)
El Salvador	2 (2.2%)
Dominican Republic	1 (1.1%)
Guatemala	1 (1.1%)
Peru	1 (1.1%)
Other	1 (1.1%)

1 Categories are not mutually exclusive and will not add to 100%

**Table 2:** Regression Analyses. Alt Text. Table illustrating the effect sizes of age, sex, caregivers’ monthly income, discrimination, parental practices and communication, and interactions on GAD-7, SDQ, and MFQ scores

	GAD-7 β (95% CI)				SDQ (Total Difficulties) β (95% CI)				MFQ β (95% CI)			
		
	Model 1	Model 2	Model 3	Model 4	Model 5	Model 6	Model 7	Model 8	Model 9	Model 10	Model 11	Model 12
Age	−0.8 (−1.5-0.01)	−0.4 (−1.2-0.4)	−0.6 (−1.4-0.1)	−0.4 (−1.2-0.3)	−0.5 (−1.2-0.2)	−0.3 (−1.0-0.5)	−0.6 (−1.4-0.2)	−0.3 (−1.1-0.5)	0.4 (−1.2-2.1)	1.0 (−0.7-2.7)	0.4 (−1.3-2.2)	0.9 (−0.9-2.8)
Sex (Male)	−0.6 (−2.8-1.6)	−0.2 (−2.6-2.2)	−0.5 (−2.9-1.8)	−0.6 (−3.1-1.8)	−2.9** (−5.1- −0.8)	−2.3 (−4.7-0.1)	−2.8* (−5.2- −0.4)	−2.7* (−5.2- −0.2)	−10.5*** (−15.4- −5.6)	−9.5*** (−14.7- −4.3)	−9.8*** (−15.2- −4.4)	−9.4** (−15.0- −3.7)
Caregivers’ Monthly Income												
$500–999	−2.5 (−5.7-0.7)	−2.6 (−6.0-0.7)	−2.2 (−5.5-1.1)	−2.9 (−6.3-0.6)	−1.1 (−4.2-1.9)	−1.9 (−5.2-1.5)	−1.4 (−4.8-2.0)	−1.2 (−4.7-2.3)	−5.3 (−12.3-1.6)	−6.3 (−13.7-0.9)	−4.8 (−12.4-2.7)	−5.0 (−12.9-2.9)
$1,000–1,999	2.0 (−1.3-5.4)	0.7 (−2.7-4.0)	1.7 (−1.7-5.1)	0.2 (−3.3-3.7)	0.9 (−2.3-4.1)	0.1 (−3.3-3.4)	1.5 (−2.1-5.0)	−0.0 (−3.5-3.5)	−0.1 (−7.3-7.1)	−2.6 (−9.9-4.7)	0.7 (−7.1-8.5)	−1.8 (−9.7-6.1)
$2000+	−2.0 (−5.3-1.3)	−2.0 (−5.8-1.3)	−1.7 (−5.2-1.7)	−2.3 (−5.9-1.2)	0.2 (−3.0-3.4)	0.2 (−3.3-3.8)	1.2 (−2.4-4.7)	0.7 (−2.9-4.3)	−4.9 (−12.2-2.3)	−5.3 (−13.0-2.5)	−2.6 (−10.4-5.3)	−3.5 (−11.6-4.6)
Racial-Ethnic Discrimination	0.2** (0.05–0.3)	0.0 (−0.3-0.4)	0.2** (−0.1-0.5)	0.0 (−0.6-0.7)	0.2*** (0.1–0.3)	−0.1 (−0.4-0.3)	0.2 (−0.1-0.6)	0.3 (−0.4-0.9)	0.5*** (0.2–0.7)	0.1 (−0.7-0.9)	0.9* (0.1–1.7)	1.3 (−0.1-2.8)
Parental Practices & Communication												
Family Conflict	1.1 (−0.2-2.3)	0.4 (−1.4-2.3)	-	-	2.6*** (1.4–3.8)	1.5 (−0.3-3.4)	-	-	5.4*** (2.7–8.1)	3.9 (−0.1-7.9)	-	-
Positive Parenting	−2.5** (−4.1- −1.0)	-	−1.7* (−3.2- −0.2)	-	−1.6* (−3.1- −0.1)	-	−2.6** (−4.2- −1.1)	-	−4.4* (−7.7- −1.1)	-	−3.6* (−7.0- −0.2)	-
Caregiver Involvement	1.6 (−0.3-3.5)	-	-	−1.1 (−3.2-0.9)	−1.3 (−3.1-0.5)	-	-	−3.0** (−5.0- −1.0)	0.4 (−3.7-4.6)	-	-	−2.4 (−7.0-2.1)
Interactions												
Family Conflict * Discrimination	-	0.1 (−0.0-0.2)	-	-	-	0.1 (−0.0-0.3)	-	-	-	0.2 (-0.1-0.5)	-	-
Positive Parenting * Discrimination	-	-	−0.0 (−0.1-0.1)	-	-	-	0.0 (−0.1-0.1)	-	-	-	−0.1 (−0.3-0.1)	-
Caregiver Involvement * Discrimination	-	-	-	0.0 (−0.1-0.2)	-	-	-	0.0 (−0.1-0.2)	-	-	-	−0.2 (−0.5-0.2)

* *p*<.05;

** *p*<.01;

*** *p*<.001

## Data Availability

The data, analytic code, and scales are not made publicly available but may be made so upon reasonable request.

## Abbreviations

SDQ: Strengths and Difficulties Questionnaire
MFQ: Mood and Feelings Questionnaire
GAD-7: Generalized Anxiety Disorder
