# Supplementary material for: Racial Discrimination and Mental Health Among Latine Adolescents: Parenting and Conflict as Potential Moderators
Source: J Racial Ethn Health Disparities. Author manuscript; Available in PMC 2026 Oct 13. (PMC13659733; doi:10.1007/s40615-026-02956-6)
Supplement: Supplementary file 1 [file NIHMS2212443-supplement-Appendix_A.docx]

Appendix A. Reliability and Validity of Study Scales

| Scale | Prior Studies | | Current Study |
| --- | --- | --- | --- |
|  | Reliability Alpha | Validity Tested | Reliability Alpha |
| Parent-Adolescent Conflict | 0.79 [21-22] | Divergent, Criterion [21-22] | 0.87 |
| Parenting Practices | 0.68–0.81 [23] | Construct [23] | 0.92 |
| Everyday Discrimination Scale | 0.87 [25-26] | Construct, criterion [25-26] | 0.92 |
| GAD-7 | 0.90 [27-28] | Convergent, Divergent [27-28] | 0.93 |
| SDQ | 0.80 [29-30] | Predictive [29-30] | 0.86 |
| MFQ | 0.85, 0.87 [31] | Content, Criterion, Predictive,  Convergent, Concurrent [31] | 0.96 |
