# Supplementary material for: Racial Discrimination and Mental Health Among Latine Adolescents: Parenting and Conflict as Potential Moderators
Source: J Racial Ethn Health Disparities. Author manuscript; Available in PMC 2026 Oct 13. (PMC13659733; doi:10.1007/s40615-026-02956-6)
Supplement: Supplementary file 2 [file NIHMS2212443-supplement-Appendix_B.docx]

Appendix B.

| Study Population Characteristics of Adolescents Excluded | | |
| --- | --- | --- |
| Characteristic | | Adolescent Count |
|  |  | n = 35 |
| Age (mean, SD) | | 14.9 (2.9) |
| Sex (Female (n, %)) | | 21 (60.0%) |
| Latino Race^1^ | |  |
|  | White (Latino/a) | 18 (51.4%) |
|  | Black-Latino/a (Afro-Latino/a) | 2 (5.7%) |
|  | Asian-Latino/a | 0 (0.0%) |
|  | Indigenous Latino/a | 6 (17.1%) |
|  | Other | 6 (17.1%) |
| Preferred Language | |  |
|  | English | 20 (57.1%) |
|  | Spanish | 14 (40.0%) |
|  | Indigenous language | 1 (2.9%) |
| Country Born | |  |
|  | USA | 25 (71.4%) |
|  | Mexico | 5 (14.3%) |
|  | Honduras | 3 (8.6%) |
|  | Venezuela | 1 (2.9%) |
|  | El Salvador | 1 (2.9%) |
| ^1^Categories are not mutually exclusive and may not add to 100%. | | |
